# Formulation and Biopharmaceutical Evaluation of Capsules Containing Freeze-Dried Cranberry Fruit Powder

**DOI:** 10.3390/plants12061397

**Published:** 2023-03-21

**Authors:** Rima Šedbarė, Valdimaras Janulis, Kristina Ramanauskiene

**Affiliations:** 1 Department of Pharmacognosy, Faculty of Pharmacy, Lithuanian University of Health Sciences, 50162 Kaunas, Lithuania; valdimaras.janulis@lsmu.lt; 2 Department of Clinical Pharmacy, Faculty of Pharmacy, Lithuanian University of Health Sciences, 50162 Kaunas, Lithuania; kristina.ramanauskiene@lsmu.lt

**Keywords:** *Vaccinium macrocarpon*, food supplement, UPLC-DAD, modified-release, chitosan

## Abstract

Cranberry fruits are an important source of anthocyanins and anthocyanidins. The aim of the present study was to investigate the effect of excipients on the solubility of cranberry anthocyanins and their dissolution kinetics as well as on the disintegration time of the capsules. Selected excipients (sodium carboxymethyl cellulose, beta-cyclodextrin and chitosan) were found to affect the solubility and release kinetics of anthocyanins in freeze-dried cranberry powder. Capsule formulations N1–N9 had a disintegration time of less than 10 min, and capsule formulation N10 containing 0.200 g of freeze-dried cranberry powder, 0.100 g of Prosolv (combination of microcrystalline cellulose and colloidal silicon dioxide), and 0.100 g of chitosan had a capsule disintegration time of over 30 min. The total amount of anthocyanins released into the acceptor medium ranged from 1.26 ± 0.06 mg to 1.56 ± 0.03 mg. Capsule dissolution test data showed that the time to release into the acceptor medium was statistically significantly longer for the chitosan-containing capsule formulations compared to the control capsules (*p* < 0.05). Freeze-dried cranberry fruit powder is a potential source of anthocyanin-rich dietary supplements, and the choice of excipient chitosan could be a suitable solution in capsule formulations providing greater anthocyanin stability and modified release in the gastrointestinal tract.

## 1. Introduction

The World Health Organization (WHO) has stated that consuming at least 400 g of vegetables, fruit, and berries per day reduces the risk of developing non-communicable diseases [1]. Berry consumption in the global market is expected to grow by around 3.5% between 2022 and 2027 as a result of increasing awareness of healthier dietary choices and the higher content of berries in processed foods [2]. The fruits of the large cranberry (*Vaccinium macrocarpon* Aiton) have been found to contain biologically active compounds whose biological effects are important for health promotion and disease prevention, which makes the research and development of the use of cranberry fruit in food products and food supplements important and promising.

Fresh cranberry fruits have been found to contain proanthocyanidins (418.8 mg/100 g) [3], anthocyanins (13.6–140 mg/100 g) [4], flavonols (20–30 mg/100 g) [5], phenolic compounds (570 mg/100 g) [6], triterpene compounds (65–122 mg/100 g) [7], vitamins (vitamins C (10.07–20.74 mg/100 g) [8], B1 (0.012 mg/100 g), B2 (0.02 mg/100 g), B3 (0.101 mg/100 g), B5 (0.295 mg/100 g), B6 (0.057 mg/100 g), and B9 (1 µg/100 g)) [9], macroelements (N (9.6–62.4 mg/100 g), P (6.0–10.8 mg/100 g), K (52.8–88.8 mg/100 g), Ca (7.2–13.2 mg/100 g), Mg (4.8–8.4 mg/100 g), and S (4.8–13.2 mg/100 g)), microelements (Fe (0.22–3.88 mg/100 g), Mn (0.058–0.51 mg/100 g), Zn (0.04–1.18 mg/100 g), Cu (0.03–0.07 mg/100 g), and B (0.03–0.11 mg/100 g)) [10], fructose (0.37–0.65 mg/100 g), glucose (3.36–4.72 mg/100 g) [8], and dietary fibers (3.6 g/100 g) [9].

Supplementing the human diet with cranberries enriches the body with natural antioxidants that can help protect the body’s systems against the development of chronic diseases [9]. Research has shown the benefits of cranberries in preventing and treating urinary tract infections [11], as well as for gastrointestinal [12,13] and oral health [14]. Clinical trials have shown positive effects of cranberry bioactive compounds in chronic diseases such as diabetes, hypercholesterolemia, and cardiovascular disease [15,16].

The use of freeze-dried berries for human consumption has recently been gaining popularity, as the qualitative and quantitative composition of the bioactive compounds in freeze-dried fruit is equivalent to that of fresh fruit [17]. The sour and astringent taste of cranberries limits the consumption of fresh fruit; thus, the fruit is processed by drying, sweetening, and juicing [18]. Freeze-dried fruits and berries are used in the food industry and can be added to various types of food such as drinks, meals, juices, and yogurts [19]. There are data in the literature on the formulation of capsules with lyophilized apple, elderberry fruits and *Morus nigra* L. fruits [20,21,22]. Although there are many nutritional supplements on the market that use cranberry fruit extracts as the active ingredient in hard capsules, the use of freeze-dried cranberry fruit or other freeze-dried botanical materials in the production of hard capsules is not often considered [23]. Cranberry fruit lyophilizate can be used in the production of functionalized food additives or food supplements of known composition and high nutritional value [24,25]. Freeze-dried fruit and berries promote long-term storage of freeze-dried food or food supplements, so the area of such products is perspective for development.

When modeling pharmaceutical formulations containing freeze-dried cranberry powder, it is important to assess the solubility, stability, and bioavailability of bioactive compounds such as anthocyanins. At low gastric pH, anthocyanins are stable flavylium cation forms, whereas when the intestinal pH increases, they are converted to less stable carbinol-base, chalcone, quinonoid-base, and anionic-quinonoid-base forms [26]. In vitro and in vivo studies have shown that, in contrast to flavonoids, anthocyanins are absorbed as glycosides [27]. Anthocyanins are rapidly absorbed, but bioavailability in blood plasma is low, amounting to only 1% of the anthocyanin intake [28,29]. In the stomach and intestines, anthocyanins are transported by transporter proteins or are metabolized [27]. Despite their low bioavailability, plasma concentrations of anthocyanins are sufficient to induce changes in signal transduction and gene expression in vivo [27]. Unabsorbed anthocyanins from the epithelial cells of the stomach and small intestines reach the large intestine, where they are actively metabolized by microorganisms, leading to an increase in the number of good intestinal bacteria *Bifidobacterium* spp., *Lactobacillus* spp. and *Actinobacteria* strains [30]. The low stability and bioavailability of anthocyanins limit their effect; therefore, strategic decisions in modeling pharmaceutical formulations are needed to achieve the targeted local supply of the active ingredient with good bioavailability and a controlled release rate [31].

One of the most widely used oral pharmaceutical forms is capsules. When modeling the contents of capsules containing cranberry lyophilizate, it is important to select appropriate excipients that would not adversely affect the absorption of anthocyanins. The excipients must be non-toxic and non-allergenic, must guarantee an effective therapeutic activity at the lowest therapeutic concentration of the active substance, and must be stable and free of interactions with the active substance [32]. Cellulose derivatives are widely used as fillers in capsule production [33]. Fillers constitute the bulk of the encapsulated content; and thus, their inherent properties should not alter the regulated disintegration time of the capsules or the dissolution kinetics of the active compounds. Solubility enhancers such as poloxamer or beta-cyclodextrins are used to improve the bioavailability of the active compounds [34,35]. Cyclodextrins taken orally are metabolized by the microbiota of the colon into the metabolites maltodextrin, maltose, and glucose, which are further metabolized into water and carbon dioxide [36]. Chitosan is used as a disintegrant in the manufacture of solid oral dosage forms, in the manufacture of controlled-release solid dosage forms, or to improve the dissolution of drugs [37]. The intrinsic properties of chitosan are useful in improving the absorption of active substances [38]. Due to the influence of excipients on pharmaceutical formulations, it is relevant to evaluate the biopharmaceutical properties of the formulated capsules with freeze-dried cranberry powder in disintegration and dissolution tests. The disintegration test for solid formulations evaluates the time taken for the formulation to lose its original form. This test helps to predict how long it would take for a pharmaceutical formulation to degrade in the human body. The amount of the active substance released into the medium at certain time intervals is highly important in the results of the dissolution test for solid dosage forms. It is, therefore, relevant to investigate the influence of fillers and polymeric materials on the dissolution kinetics of the active compounds in cranberries. The aim of the present study was to investigate the effect of excipients on the solubility of cranberry anthocyanins and their dissolution kinetics as well as on the disintegration time of the capsules.

## 2. Results

**Figure 1:**
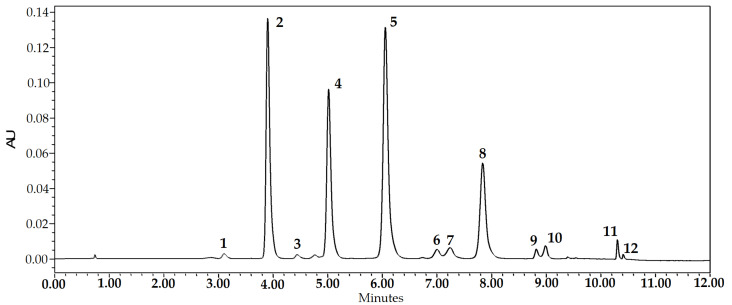
Chromatogram profile of anthocyanins and anthocyanidins in the extract of large cranberry fruit samples at 520 nm. 1—delphinidin-3-galactoside; 2—cyanidin-3-galactoside; 3—cyanidin-3-glucoside; 4—cyanidin-3-arabinoside; 5—peonidin-3-galactoside; 6—peonidin-3-glucoside; 7—malvidin-3-galactoside; 8—peonidin-3-arabinoside; 9—cyanidin; 10—malvidin-3-arabinoside; 11—peonidin; 12—malvidin.

The aim of the first stage of the study was to determine the qualitative and quantitative composition of anthocyanins and anthocyanidins in freeze-dried cranberry fruit samples. Freeze-dried cranberry powder extracts are multicomponent matrices, and the determination of their composition requires state-of-the-art analytical tools to ensure rapid, selective, and reproducible evaluation of bioactive compounds. Anthocyanins (delphinidin-3-galactoside, cyanidin-3-galactoside, cyanidin-3-glucoside, cyanidin-3-arabinoside, peonidin-3-galactoside, peonidin-3-glucoside, malvidin-3-galactoside, peonidin-3-arabinoside, and malvidin-3-arabinoside) and anthocyanidins (cyanidin, peonidin, and malvidin) were identified and quantified by ultra-high-performance liquid chromatography (UHPLC). The chromatogram of the anthocyanin group compounds detected in the freeze-dried cranberry fruit extract is presented in Figure 1.

**Figure 2:**
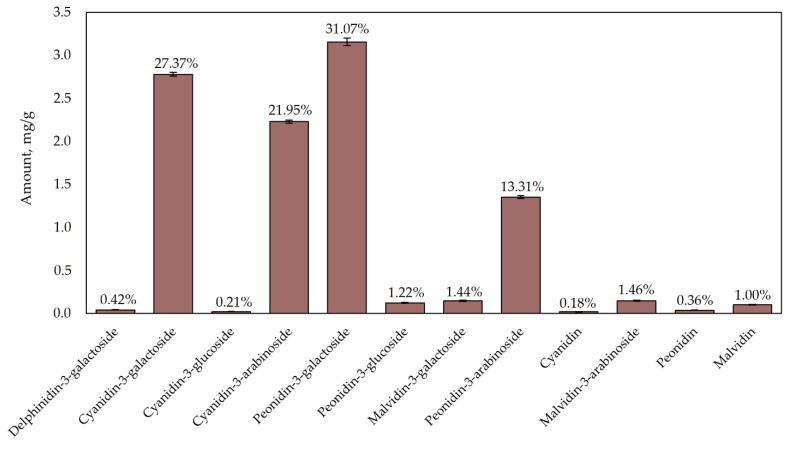
Concentration of individual anthocyanins in ethanol extracts obtained from the fruit of the ‘Dižbrūklene’ cranberry cultivar.

A graph of the quantitative and percentage composition of the compounds identified in the freeze-dried cranberry fruit samples is presented in Figure 2. Four compounds were predominant in the cranberry fruit samples, accounting for 94% of the total anthocyanins identified. In fruit samples of the cranberry cultivar ’Dižbrūklene’, the highest content of a single anthocyanin found (3.16 ± 0.04 mg/g) was that of peonidin-3-galactoside, accounting for 31.07% of the total anthocyanin content. The detected amount of cyanidin-3-galactoside was 2.78 ± 0.02 mg/g, accounting for 27.37% of the total anthocyanin content, while the detected amount of cyanidin-3-arabinoside was 2.23 ± 0.02 mg/g, accounting for 21.95% of total anthocyanin content. The detected amount of peonidin-3-arabinoside was 1.35 ± 0.02 mg/g, accounting for 13.31% of total anthocyanin content.

Other identified anthocyanins and anthocyanidins accounted for 6% of the total anthocyanin content. Samples of freeze-dried cranberry fruit were found to contain 0.15 ± 0.01 mg/g of malvidin-3-arabinoside, 0.15 ± 0.01 mg/g of malvidin-3-galactoside, 0.12 ± 0.01 mg/g of peonidin-3-glucoside, 0.10 ± 0.01 mg/g of malvidin, 0.04 ± 0.00 mg/g of delphinidin-3-galactoside, 0.04 ± 0.00 mg/g of peonidin, 0.02 ± 0.00 mg/g of cyanidin-3-glucoside, and 0.02 ± 0.00 mg/g of cyanidin.

**Figure 3:**
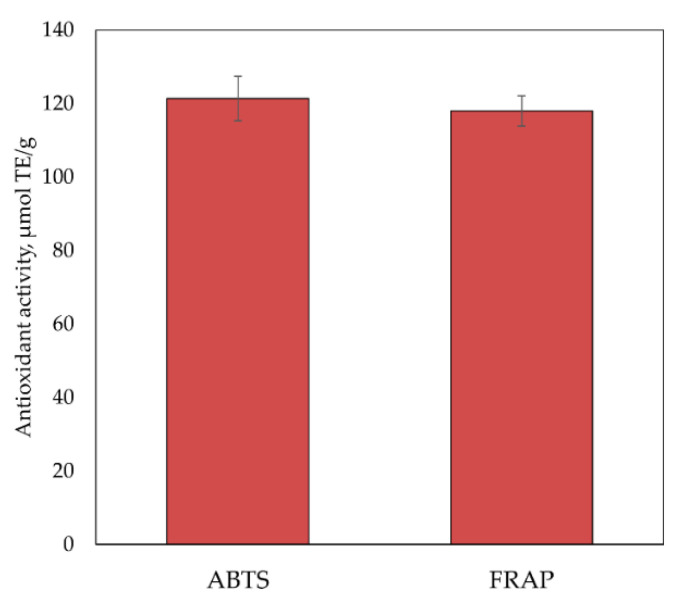
Antioxidant activity of cranberry ethanol extracts.

The antioxidant activity of the multi-component matrix complex of freeze-dried cranberry fruit extracts was determined in vitro by applying the antioxidant ABTS and FRAP methods. The results of the antioxidant activity assays of the cranberry extracts are presented in Figure 3. The evaluation of the antioxidant activity of the freeze-dried cranberry fruit extracts showed that their anti-radical activity was 121.35 ± 6.07 µmol TE/g, as assessed by the ABTS method. The reducing activity of the freeze-dried cranberry fruit extracts, assessed by the FRAP method, was found to be 118.00 ± 4.13 µmol TE/g.

In the next stage of the study, solid gelatin capsules of different compositions with freeze-dried cranberry fruit powder were formulated and biopharmaceutical evaluation of the prepared capsules was conducted. Ten capsules of different compositions (N1–N10) were formulated in the study, and the compositions of these capsules are presented in Table 1. Based on the anthocyanin content of the freeze-dried cranberry powder, we chose to use 0.200 g of freeze-dried cranberry powder per hard gelatin capsule. The encapsulated mixture contained a combination of microcrystalline cellulose and colloidal silicon dioxide (Prosolv) as a filler. Capsule formulations N2 to N4 contained between 0.025 g and 0.100 g of sodium carboxymethylcellulose, capsule formulations N5 to N7 contained between 0.025 g and 0.100 g of beta-cyclodextrin, and capsule formulations N8 to N10 contained between 0.025 g and 0.100 g of chitosan. In the manufacture of the encapsulated compound, sodium carboxymethylcellulose and chitosan were used as ingredients prolonging the release of the active substance. Beta-cyclodextrin was chosen as a solubility and stability enhancer for the active compounds.

The quality of the produced cranberry lyophilizate capsules was assessed based on the results of mass uniformity, disintegration time, and dissolution tests. The data presented in Table 1 on the predicted theoretical and the observed mass of the encapsulated mixture in the capsule show that the capsules were similar in terms of mass uniformity (percentage deviation < 7.5%). The selected active ingredients and excipients and their quantities ensured accurate dosing of the encapsulated mixture into the capsules.

**Figure 4:**
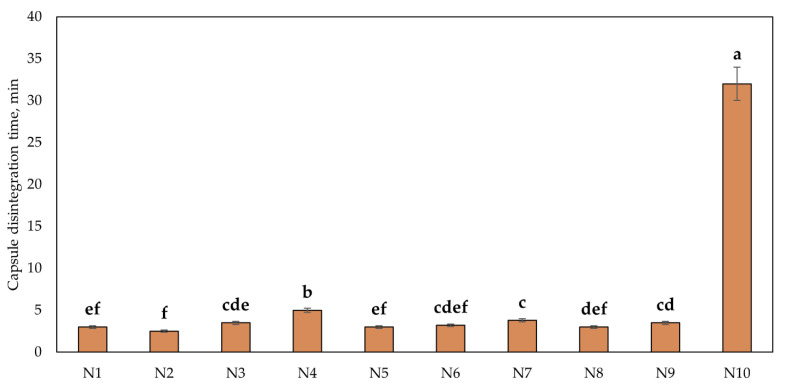
Results of the capsule disintegration test. Different letters indicate significant differences (*p* < 0.05) between the tested capsule formulations, and capsule formulations with same letter were not significantly different (*p* > 0.05). The compositions of the capsules (N1–N10) are described in Table 1.

The capsule quality parameter—the disintegration time—indicates how long it takes for a capsule to fully disintegrate. This parameter is important because the release of the active compounds does not start until the capsule has disintegrated, and excipients can significantly alter the disintegration time. The disintegration times of the ten N1–N10 capsules formulated in the study are shown in Figure 4. Cranberry lyophilizate powder-containing capsules N1–N9 had a disintegration time of less than 10 min. Increasing the weight of the encapsulated contents to 0.400 g (capsules N4, N7, and N10) resulted in a statistically significant increase in the disintegration time. The disintegration time of capsules with freeze-dried cranberry powder weighing 0.400 g was most affected by chitosan. The disintegration time of capsule N10 containing 0.200 g of freeze-dried cranberry powder, 0.100 g of Prosolv, and 0.100 g of chitosan was longer than 30 min.

One of the most important tests for assessing the quality of capsules is the determination of the dissolution kinetics of the active compounds. To determine the dissolution kinetics of the formulated capsules, a dissolution test was applied using a paddle-type dissolution apparatus, the mechanism of action of which mimics the physiological conditions of the body in vitro. Four cranberry fruit anthocyanins, cyanidin-3-galactoside, cyanidin-3-arabinoside, peonidin-3-galactoside, and peonidin-3-arabinoside, were identified in the acceptor medium during the dissolution test. Other anthocyanins and anthocyanidins present in the cranberry fruit samples could not be detected because their concentrations in the acceptor medium were below the limit of detection (LOD) or limit of quantification (LOQ).

**Figure 5:**
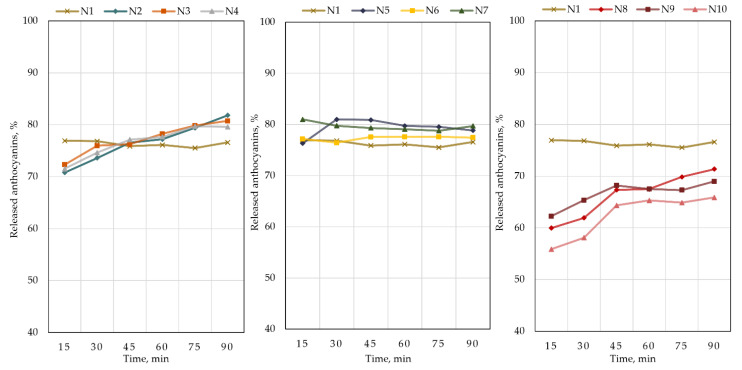
Results of the dissolution test of cranberry lyophilizate-containing capsules of different compositions. The compositions of the capsules (N1–N10) are described in Table 1.

The results of the dissolution test on cranberry lyophilizate-containing capsules N1–N10 are shown in Figure 5. The kinetic plots of the dissolution test (Figure 5) show the amount of anthocyanins released in the acceptor media from the formulated capsules (N1–N10) at different time points. In the graphs, the dissolution test results of capsule formulations N1 containing freeze-dried cranberry powder and Prosolv are shown as a comparison sample. The release of active compounds from capsules N1 was most intense within the 15 min period. There was no statistically significant difference between the levels of active compounds in the acceptor medium at baseline and after 90 min of testing. Mathematical analysis of the kinetic profile of anthocyanins showed that the regression coefficient of the Higuchi model for formulation N1 was 0.339. Capsule formulations N2, N3, and N4 released, respectively, 70.8%, 72.3%, and 71.5% of anthocyanins into the acceptor medium within 15 min. Thereafter, the release of the active compounds was slower. After 90 min, 81.8%, 80.7%, and 79.6% of anthocyanins were released into the acceptor medium from capsule formulations N2, N3, and N4, respectively. The regression coefficients of the Higuchi model of capsule formulations N2, N3, and N4 were 0.984, 0.970, and 0.959, respectively. The dissolution test data for capsules N2–N4 showed that the amount of anthocyanins released from these sodium carboxymethylcellulose-containing formulations within 15 min was not statistically significantly different from the amount released from the control formulation N1.

Capsule formulations N5, N6, and N7 released, respectively, 76.3%, 77.1%, and 80.9% of the anthocyanins into the acceptor medium within 15 min. Anthocyanins dissolved most intensively in the 15 min interval; the dissolution kinetics of anthocyanins was not intense at the following test points (*p* > 0.05). After 90 min, the amount of anthocyanins released into the acceptor medium by capsule formulations N5, N6, and N7 remained similar at 78.8%, 77.4%, and 79.7%, respectively. The Higuchi regression coefficients of formulations N5, N6, and N7 were 0.106, 0.345, and 0.550, respectively. The dissolution kinetics profile of capsules N5–N7 was similar to that of the comparator sample N1; the addition of beta-cyclodextrins did not statistically significantly (*p* > 0.05) improve the solubility of the active compounds, with only an increase of approximately 3% in the solubility of the anthocyanins in the acceptor medium.

Capsule formulations N8, N9, and N10 released, respectively, 59.9%, 62.2%, and 55.9% of the anthocyanins into the acceptor medium within 15 min. During the dissolution test, the amount of anthocyanins released into the acceptor medium from capsule formulations N8, N9, and N10 gradually increased and after 90 min was, respectively, 71.3%, 69.0%, and 65.9%. The Higuchi regression coefficients of formulations N8, N9, and N10 were 0.957, 0.798, and 0.859, respectively. Chitosan-containing capsules N8–N10 showed a statistically significant prolongation of the release of anthocyanins into the acceptor medium compared to the comparator sample N1 (*p* < 0.05).

**Figure 6:**
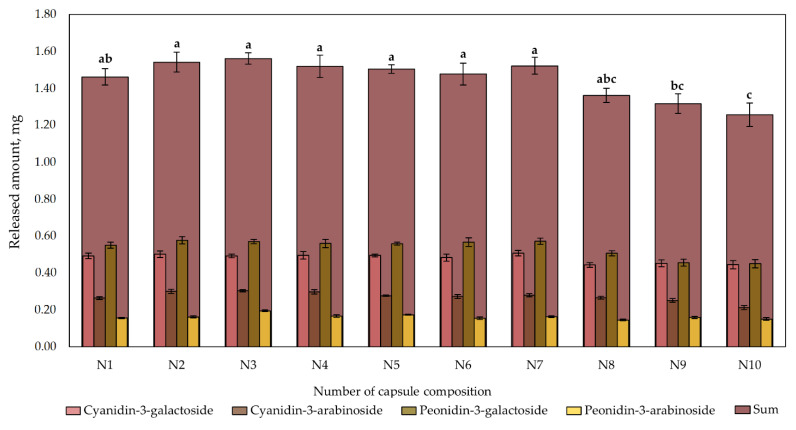
Content of the individual anthocyanins and their sum released from cranberry lyophilizate-containing capsules of different composition after 90 min. The compositions of the capsules (N1–N10) are described in Table 1. Different letters indicate significant differences (*p* < 0.05) between sum amounts of anthocyanins in the tested capsule formulations, and capsule formulations with same letter were not significantly different (*p* > 0.05).

The results of the quantitative composition of cranberry lyophilizate-containing capsules N1–N10 after 90 min obtained during the dissolution test are shown in Figure 6. The total amount of anthocyanins released into the acceptor medium varied from 1.26 ± 0.06 mg to 1.56 ± 0.03 mg. There was no statistically significant difference in total anthocyanin content released from capsule formulations N1–N8 after 90 min (*p* > 0.05). The total amount of anthocyanins released after 90 min from capsule formulations N9 and N10 was statistically significantly lower than that released from capsule formulations N1–N7. During the dissolution test, extended-release capsule formulations N9 and N10 released by, respectively, up to 0.25 and 0.3 mg less anthocyanins than the other formulations did.

The amount of peonidin-3-galactoside comprised 34.57–38.37%, the amount of cyanidin-3-galactoside—31.21–35.41%, the amount of cyanidin-3-arabinoside—16.87–19.58%, and the amount of peonidin-3-arabinoside—10.49–12.51% of the sum of identified anthocyanins during the solubility test. The amount of anthocyanins released into the acceptor medium of 0.1 N hydrochloric acid in aqueous solution from formulations N1–N10 was 18% to 34% lower than the amount of anthocyanins detected in the ethanol extract of freeze-dried cranberry powder. It should be noted that, on average, approximately 65% of anthocyanidin arabinosides and approximately 85% of anthocyanidin galactosides were released from the capsule formulations into the acceptor medium of 0.1 N hydrochloric acid in aqueous solution.

This study showed that the selected excipients altered the kinetics of the release of anthocyanins from freeze-dried cranberry powder. Capsule formulations N8–N10 containing the excipient chitosan showed a statistically significant prolongation of the release time into the acceptor medium. Capsule formulation N10 with 0.100 g of chitosan prolonged the disintegration time of the capsule by >30 min.

## 3. Discussion

Anthocyanin-containing fruits and vegetables in the human diet reduce the development of inflammatory processes in the body caused by oxidative stress and may improve the composition of the gut microbiota [28]. In the United States, the estimated daily intake of anthocyanins is 12.5 mg [39]. Guo et al. found that daily intake of 7.5 mg of anthocyanins from fruit reduced the risk of developing type 2 diabetes [40]. In this study, we found that freeze-dried cranberry powder contained 10.16 ± 0.12 mg/g of anthocyanins. Brown et al. and Xue et al. investigated the anthocyanin content of cranberry lyophilizate raw material and found a lower anthocyanin content (accordingly, 2.81–7.98 mg/g and 7.25 mg/g) than that found in our study [41,42]. The total anthocyanin content of fresh cranberry samples in a study by Narwojsz et al. was by approximately 25 times lower than the total anthocyanin content of freeze-dried cranberry raw material found in our study [43]. In our study, freeze-dried cranberry powder was used for pharmaceutical formulations to enrich them with the bioactive compounds found in cranberry fruit. The freeze-dried cranberry powder formulations used in this study each contained 0.200 g of freeze-dried cranberry powder, equivalent to 2 mg of anthocyanins. The freeze-dried cranberry powder used in the studied formulations retains the bioactive properties of natural cranberries, but the amount of anthocyanins per capsule would not provide the recommended daily intake (12.5 mg); therefor, a higher number of capsules with 0.200 g of freeze-dried cranberry powder should be consumed per day.

Hard gelatin capsules are a very common and unique form of capsule, consisting of a cap and a body [44]. Capsule disintegration and solubility are influenced by the properties of the mixture of active compounds and excipients. The excipient Prosolv (a combination of microcrystalline cellulose and colloidal silicon dioxide) was used in capsule fillings due to its characteristic properties: uniformity of content, higher density, low moisture content, flowability, and spreadability [45,46]. In this study, we found that the capsule formulation N1 consisting of 0.200 g of freeze-dried cranberry powder and 0.100 g of Prosolv released 76.9% of the anthocyanins within 15 min (Figure 5). The amount of anthocyanins released by the capsule formulation into the acceptor medium remained statistically significantly constant throughout the study. During the study, the reasons why less than 82% of the anthocyanins were released from capsule formulations N1–N10 into the acceptor medium could have been the following: interaction of the freeze-dried cranberry powder with the excipients [47], lower solubility in the aqueous medium [48], and biodegradation in the acceptor medium [49].

The interaction between anthocyanins and polysaccharide excipients is important in stabilizing anthocyanins and protecting them from degradation in the biological environment as well as in modifying the release of anthocyanins from capsule formulations [31]. The polysaccharide sodium carboxymethylcellulose is a cellulose derivative which is highly soluble in water [50]. Sodium carboxymethylcellulose has stabilizing, emulsifying, and viscosity-modifying properties [50,51] and may, therefore, be used as a release-modifying excipient for the active compounds of the capsules [46]. The release kinetics of the N2–N4 capsule formulations tested in this study might have been influenced by the effect of sodium carboxymethylcellulose on the viscosity of the medium, which resulted in a gradual increase in the release of anthocyanins into the acceptor medium by approximately 10% from 15 min to 90 min, and the amount of anthocyanins released into the acceptor medium was greater than that of the control capsule formulation N1.

Freeze-dried cranberry powder capsule formulations N5–N7 were formulated with beta-cyclodextrin used as an excipient to improve solubility and stability [52]. Beta-cyclodextrin is a cyclic oligosaccharide with supramolecular structures that carry out chemical reactions involving intermolecular interactions where no covalent bonds are formed between interacting molecules, ions, and radicals [53]. The ability of beta-cyclodextrin to form complexes with various organic compounds helps to modify the apparent solubility of the molecule and to increase the stability of the compound under the exposure to light, heat, and oxidizing conditions [52]. In this study, the solubility of capsule formulations with beta-cyclodextrin (N5–N7) improved quantitatively compared to the control formulation, but these changes were not statistically significant (*p* > 0.05) (Figure 6). This result could be due to the relatively low solubility of beta-cyclodextrin in water at low pH. Shen et al. have argued that it is important to select the appropriate ratio of anthocyanins to beta-cyclodextrin to increase the solubility of the compounds [31]. In our experimental study, modeling pharmaceutical formulations with different ratios of freeze-dried cranberry powder to beta-cyclodextrin did not improve solubility. Fernandes et al. investigated the stability and release kinetics of the anthocyanin–beta-cyclodextrin complex in blackberry puree under gastrointestinal conditions in vitro [54]. They found that anthocyanins were stable at acidic gastric pH, but decomposed within 15 min once in the intestine, where the pH is higher: 55% of the anthocyanins in the complex with beta-cyclodextrin decomposed, compared to 71% of the anthocyanins in the anthocyanin control solution [54]. The obtained results showed the ability of beta-cyclodextrin to reduce the degradation rate of anthocyanins under simulated gastrointestinal conditions, but such a complex does not provide a modified release, which is more protective of anthocyanins against degradation [55]. Given that anthocyanins are more stable in the acidic medium than in intestinal fluids, it is expedient to look for excipients to slow their degradation and improve their absorption.

The chitosan used in capsule formulations N8–N10 is a polysaccharide derived from the deacetylation of chitin, a polysaccharide widely distributed in nature (in crustaceans, insects, and fungi) [56]. Chitosan is used as a natural, biodegradable, antibacterial, gelling, and active substance release-modifying excipient [31,57]. Positively charged chitosan hydrochloride may form a polyelectrolyte film encapsulating charged compounds, thereby slowing their release in the digestive tract [58,59]. The extended release of formulations N8–N10 in this study can be explained by the anthocyanins forming a flavylium cation in the acidic acceptor medium, which possibly interacts with the polymeric structures of the chitosan and enters the gel structures formed by the chitosan, thus prolonging the release of anthocyanins into the acceptor medium [31]. As the chitosan content increased in the tested capsules (N8 < N9 < N10), a higher proportion of the anthocyanins was incorporated into chitosan complexes, resulting in lower amounts of anthocyanins in the acceptor medium. Thus, the chitosan–anthocyanin complex potentially increases anthocyanin stability and bioavailability [31]. He et al. investigated the release of the nanoparticles of the blueberry anthocyanin–chitosan complex from dialysis membrane bags under gastrointestinal conditions [60]. They found that after 120 min, the amount of anthocyanins released into the acceptor medium from the anthocyanin–chitosan complex was lower (47.73%) than the amount of anthocyanins released from the free anthocyanin solution (68.53%) [60]. Chitosan has the ability to stabilize anthocyanins in nanoparticle formulations and to slow their degradation in the gastrointestinal tract, and the slow release can reduce the degradation of the bioactive compounds and improve their bioavailability and absorption in the gastrointestinal tract [60,61].

The instability and low bioavailability of anthocyanins require strategic solutions to achieve targeted delivery of anthocyanins with good bioavailability and controlled release rates [31]. Pharmaceutical formulations stabilizing anthocyanins and modifying their release kinetics may be important in delivering higher amounts of unabsorbed and unmetabolized anthocyanins to the colon, where they are metabolized by gut bacteria [62]. Based on bioavailability and absorption data, most of the anthocyanins consumed are actively metabolized and converted into small metabolites by gut bacteria such as *Bifidobacterium* spp. and *Lactobacillus* spp. [30]. Liu et al. found that administration of cranberry anthocyanin extracts increased the growth of the bacteria *Lachnoclostridium*, *Roseburia*, and the *Clostridium innocuum* group, and decreased the growth of the strains *Rikenella* and *Rikenellaceae* in mice [63]. Anhê et al. found that mice fed 200 mg/kg cranberry extract for 8 weeks had an increased population of *Akkermansia* spp. in their gut microbiota [64]. Morato et al. conducted a randomized controlled trial in 11 healthy adults and found that a cranberry diet with a daily intake of 30 g of freeze-dried cranberry powder resulted in an increase in *Bacteroidetes* and a decrease in *Firmicutes* bacterial populations in healthy adults after 5 days of treatment, when compared to a placebo powder diet [13]. Good intestinal bacteria are important for improving the metabolism of phenolic compounds, enhancing intestinal barrier function, increasing mucus secretion, producing short-chain fatty acids, and regulating lipid metabolism [65]. Freeze-dried cranberry fruit powder showed antioxidant activity. The results of the dissolution test showed that cranberry lyophilizate capsules could be a source of natural antioxidants. Freeze-dried cranberry powder could be used in the development of prebiotic formulations, but it is important to select the appropriate excipients for such formulations to ensure the targeted metabolism of anthocyanins in the colon.

This study of capsules with lyophilized fruit, like many other similar studies, is focused on disease prevention and healthier nutrition in order to provide quality cranberry fruit products to increase the amount of fruit and cranberry products consumed in modern society. On the other hand, in contrast to other researchers, we aim to use whole cranberry fruit lyophilizate, but not the processed cranberry extract used by many researchers [23]. This helps preserve the natural and complete composition of cranberry fruit’s biologically active compounds for benefits for health.

## 4. Materials and Methods

### 4.1. Preparation of Freeze-Dried Cranberry Powder

For the experiment, fruits of large cranberry (*Vaccinium macrocarpon* Aiton) cultivar ‘Dižbrūklene’ were collected on 30 September 2021, in the collection of the National Botanic Garden (Salaspils, Latvia) (56°51′55.4″ N 24°21′36.9″ E). Fresh cranberry fruit samples were frozen at −20 °C in a freezer. Subsequently, the fruits were frozen at −60 °C in an ultra-low temperature freezer (CVF330/86, ClimasLab SL, Barcelona, Spain). The fruits were freeze-dried at a condenser temperature of −85 °C and a pressure of 0.01 mbar in a lyophilizer (Zirbus, Zirbus Technology GmbH, Bad Grund, Germany). Freeze-dried cranberry fruit powder (particle size approximately 100 µm) was prepared by grinding lyophilized fruits in an electric mill (Retsch GM 200, Retsch GmbH, Hahn, Germany). Loss on drying was estimated using the method described in the European Pharmacopoeia [66].

### 4.2. Reagents

Acetonitrile and hydrochloric acid were purchased from Sigma-Aldrich (Steinheim, Germany). Ethanol 96% (*v*/*v*) was purchased from Stumbras AB (Kaunas, Lithuania). Formic acid was purchased from Merck (Darmstadt, Germany). The standard anthocyanins and anthocyanidins, including peonidin chloride, peonidin-3-galactoside, peonidin-3-arabinoside, peonidin-3-glucoside, cyanidin chloride, cyaniding-3-galactoside, cyaniding-3-glucoside, cyaniding-3-arabinoside, malvidin chloride, malvidin-3-galactoside, malvidin-3-arabinoside, and delphinidin-3-galactoside were purchased from Extrasynthese (Genay, France). Excipients (sodium carboxymethylcellulose, beta-cyclodextrin, chitosan (deacetylation grade ≥ 75%, medium molecular weight)) was purchased from Sigma-Aldrich (Steinheim, Germany); PROSOLV SMCC ^TM^ 50 (Penwest, UK). Deionized water was prepared by a Milli-Q water purification system (Milli-Q^®^, Millipore, MA, USA).

### 4.3. Extraction Procedure

During the study, 1 g of freeze-dried cranberry powder (exact weight) was weighed and extracted with 20 mL of 70% (*v*/*v*) ethanol solution containing 1% hydrochloric acid at 80 kHz and 565 W for 15 min in an ultrasonic bath (Elmasonic P, Elma Schmidbauer GmbH, Singen, Germany) at room temperature. The extract of freeze-dried cranberry powder was filtered into a 20 mL volumetric flask. Extraction of freeze-dried cranberry powder was performed three times.

### 4.4. Spectrophotometric Study

An ABTS+ (radical cation decolorization study) antioxidant activity determination was performed using the methodology introduced by Re et al. [67] and described and modified by Raudone et al. [68]. A volume of 3 mL of the ABTS∙+ solution (absorbance 0.800 ± 0.02) was mixed with 5 µL of the ethanol cranberry extract. The samples were kept in the dark for 30 min, then the decrease in absorbance was measured at 734 nm. The antiradical activity of the ethanolic cranberry extract was calculated from the standard curve (𝑦 = 0.00004𝑥 − 0.0823; R^2^ = 0.999) which was prepared using standard Trolox solutions of 2000–11,000 μmol/L concentration.

The FRAP (ferric reducing antioxidant power) assay was performed by using methodology described by Raudone et al. [69]. A volume of 3 mL of the prepared FRAP reagent was mixed with 5 µL of ethanol cranberry extract. After 30 min, the absorbance was read at 593 nm using a UV–vis spectrophotometer. The reducing antioxidant power of the ethanolic cranberry extract was calculated from the standard curve (𝑦 = 0.00006𝑥 − 0.0235; R^2^ = 0.995) which was prepared from standard Trolox solutions (2000–11,000 μmol/L).

### 4.5. Encapsulation Process

Number 0 hard gelatin capsules were used in this study. The compositions of the capsule fillings are described in Table 1. The capsule filler powder was prepared by simply mixing a freeze-dried cranberry powder with an excipient. The capsules were prepared using the manual capsule filling machine (Capsuline, Davie, FL, USA).

### 4.6. Test of the Uniformity of Mass of Single-Dose Preparations

The determination of mass uniformity was performed based on the European Pharmacopoeia [70]. The tested capsules were weighed, and the mean weight of 1 capsule was determined. Next, each capsule was carefully opened, and the content was removed. The emptied capsule was re-weighed. The mass of the content was the difference between the two weighings. The percentage deviation was set to ±7.5% for capsules with a mass greater than 300 mg.

### 4.7. In vitro Disintegration Test

The capsule disintegration test was performed according to the methodology described in the European Pharmacopoeia [71]. The disintegration time was determined using the device C-MAG HS7 (IKA^®^-Werke GmbH & Co, Staufen, Germany). The disintegration medium was 0.1 N hydrochloric acid solution, temperature 37 ± 0.50 °C, observed for 30 min.

### 4.8. In Vitro Dissolution Test

In vitro dissolution test of all freeze-dried cranberry fruit powders contained in hard gelatin capsules was performed using a dissolution tester (Sotax AT 7 smart, SOTAX AG, Allschwil, Switzerland). The acceptor medium was 250 mL of 0.1 N hydrochloric acid solution. The samples were taken after 15, 30, 45, 60, 75, and 90 min. The test was conducted at a rotational speed of 100 rpm and the temperature of 37 ± 0.5 °C. During the analysis, 2.5 mL of the sample was withdrawn from the dissolution media and replaced by 2.5 mL of fresh media solution. The kinetic release regression coefficients were found by Higuchi model [72].

### 4.9. UPLC-DAD Analysis

Prior to the chromatographic analysis, the test samples were filtered through filters with 0.45 µm pore size (CHROMAFIL^®^ Xtra PTFE45/25, 0.45 µm, Macherey–Nagel, GmbH and Co. KG, Dueren, Germany). Anthocyanins and anthocyanidins were estimated using the methodology described by Vilkickyte et al. [73]. The compounds were quantified using an Ultra-High-Performance Liquid Chromatography system (Waters ACQUITY UPLC, Milford, MA, USA) with a photodiode array detector (ACQUITY UPLC PDA eλ, Milford, MA, USA) and an ACE C18 reversed phase (100 × 2.1 mm, 1.7 µm particles) column (An Avantor ACE, ACT, Aberdeen, UK). The injection volume was 1 μL, and the flow rate was 0.5 mL/min. Mobile phases were 100% acetonitrile (*v*/*v*) (A) and aqueous 10% formic acid solution (*v*/*v*) (B). The elution gradients were as described: 0.0–2.0 min, 5-5% B; 2.0–7.0 min, 5–9% B; 7.0–9.0 min, 9–12% B; 9.0–10.0 min, 12–25% B; 10.0–10.5 min, 25–80% B; 10.5–11.0 min, 80–80% B; and 11.0–12.0 min, 80–5% B. The analysis was performed at the temperature of 30 °C. Anthocyanins were monitored at 520 nm by Empower software (Waters, Milford, MA, USA). The concentration of anthocyanins was calculated using the calibration curves constructed from standards.

### 4.10. Data Analysis

Data were presented as mean ± standard deviation, and in vitro simulation was conducted in triplicate. Statistical analysis was performed using SPSS Statistics 21 (IBM, Armonk, NY, USA). Kruskal–Wallis one-way ANOVA testfor multiple comparisons was used to evaluate the difference in the amounts of anthocyanins between the test samples. Graphs were generated with Microsoft Excel (Microsoft, Redmond, WA, USA). The amounts of compounds in different samples were considered significantly different if *p* < 0.05.

## 5. Conclusions

The study determined the effect of excipients on the dissolution kinetics of anthocyanins in hard gelatin capsules with freeze-dried cranberry powder. The excipient chitosan allows us to obtain modified-release hard gelatin capsules. Chitosan also increased the disintegration time of the formulated capsules by >30 min when its amount reached 25% of the capsule weight. It is promising to study the interactions between chitosan and other excipients such as slip and solubility improvers in order to improve the properties of capsules with freeze-dried cranberry powder. Chitosan-containing capsules with freeze-dried cranberry powder could be formulated by developing them as modified-release capsules, which could provide higher amounts of unabsorbed and unmetabolized anthocyanins in the large intestine, thus modulating the composition of gut bacteria that metabolize them. Additionally, this study can be extended by investigating the release kinetics of other phenolic compounds from capsule formulations with freeze-dried cranberry.

## Figures and Tables

**Table 1:** Compositions of the cranberry lyophilizate-containing capsules.

No	CL, g	PS, g	NaCMC, g	BCD, g	CT, g	TCM, g	CM, g
N1	0.200	0.100	-	-	-	0.300	0.301 ± 0.001
N2	0.200	0.100	0.025	-	-	0.325	0.326 ± 0.001
N3	0.200	0.100	0.050	-	-	0.350	0.352 ± 0.002
N4	0.200	0.100	0.100	-	-	0.400	0.400 ± 0.002
N5	0.200	0.100	-	0.025	-	0.325	0.324 ± 0.001
N6	0.200	0.100	-	0.050	-	0.350	0.350 ± 0.001
N7	0.200	0.100	-	0.100	-	0.400	0.399 ± 0.001
N8	0.200	0.100	-	-	0.025	0.325	0.324 ± 0.002
N9	0.200	0.100	-	-	0.050	0.350	0.349 ± 0.002
N10	0.200	0.100	-	-	0.100	0.400	0.401 ± 0.001

No—capsule composition number; CL—cranberry lyophilizate; PS—Prosolv; NaCMC—sodium carboxymethylcellulose; BCD—beta-cyclodextrin; CT—chitosan; TCM—theoretical mass of capsule filling; CM—mass of capsule filling.

## Data Availability

All data generated during this study are included in this article.

## References

[1] World Health Organisation (WHO) Healthy Diet. https://www.who.int/news-room/fact-sheets/detail/healthy-diet#:~:text=At%20least%20400%20g%20.

[2] Mordor Intelligence Fresh Berries Market—Growth, Trends, COVID-19 Impact, and Forecasts (2023–2028). https://www.mordorintelligence.com/industry-reports/fresh-berries-market.

[3] Gu L., Kelm M.A., Hammerstone J.F., Beecher G., Holden J., Haytowitz D., Gebhardt S., Prior R.L. (2004). Concentrations of Proanthocyanidins in Common Foods and Estimations of Normal Consumption. J. Nutr..

[4] Pappas E., Schaich K.M. (2009). Phytochemicals of Cranberries and Cranberry Products: Characterization, Potential Health Effects, and Processing Stability. Crit. Rev. Food Sci. Nutr..

[5] Neto C.C. (2007). Cranberry and Blueberry: Evidence for Protective Effects against Cancer and Vascular Diseases. Mol. Nutr. Food Res..

[6] Zuo Y., Wang C., Zhan J. (2002). Separation, Characterization, and Quantitation of Benzoic and Phenolic Antioxidants in American Cranberry Fruit by GC−MS. J. Agric. Food Chem..

[7] Kondo M., MacKinnon S.L., Craft C.C., Matchett M.D., Hurta R.A.R., Neto C.C. (2011). Ursolic Acid and Its Esters: Occurrence in Cranberries and Other Vaccinium Fruit and Effects on Matrix Metalloproteinase Activity in DU145 Prostate Tumor Cells: Anti-Tumor Activity and Content of Ursolic Acid from Vaccinium Fruit. J. Sci. Food Agric..

[8] Oszmiański J., Kolniak-Ostek J., Lachowicz S., Gorzelany J., Matłok N. (2017). Phytochemical Compounds and Antioxidant Activity in Different Cultivars of Cranberry (*Vaccinium Macrocarpon* L). J. Food Sci..

[9] Nemzer B.V., Al-Taher F., Yashin A., Revelsky I., Yashin Y. (2022). Cranberry: Chemical Composition, Antioxidant Activity and Impact on Human Health: Overview. Molecules.

[10] Karlsons A., Osvalde A., Čekstere G., Pormale J. (2018). Research on the Mineral Composition of Cultivated and Wild Blueberries and Cranberries. Agron. Res..

[11] Vostalova J., Vidlar A., Simanek V., Galandakova A., Kosina P., Vacek J., Vrbkova J., Zimmermann B.F., Ulrichova J., Student V. (2015). Are High Proanthocyanidins Key to Cranberry Efficacy in the Prevention of Recurrent Urinary Tract Infection?. Phytother. Res..

[12] Howell A.B. (2020). Clinical Evidence Supporting Cranberry as a Complementary Approach to Helicobacter Pylori Management. Food Front..

[13] Rodríguez-Morató J., Matthan N.R., Liu J., de la Torre R., Chen C.-Y.O. (2018). Cranberries Attenuate Animal-Based Diet-Induced Changes in Microbiota Composition and Functionality: A Randomized Crossover Controlled Feeding Trial. J. Nutr. Biochem..

[14] Philip N., Walsh L. (2019). Cranberry Polyphenols: Natural Weapons against Dental Caries. Dent. J..

[15] Masnadi Shirazi K., Shirinpour E., Masnadi Shirazi A., Nikniaz Z. (2021). Effect of Cranberry Supplementation on Liver Enzymes and Cardiometabolic Risk Factors in Patients with NAFLD: A Randomized Clinical Trial. BMC Complement. Med. Ther..

[16] Thimóteo N.S.B., Scavuzzi B.M., Simão A.N.C., Dichi I. (2017). The Impact of Cranberry (*Vaccinium Macrocarpon*) and Cranberry Products on Each Component of the Metabolic Syndrome: A Review. Nutrire.

[17] Ciurzyńska A., Lenart A. (2011). Freeze-Drying—Application in Food Processing and Biotechnology—A Review. Pol. J. Food Nutr. Sci..

[18] Michalska-Ciechanowska A., Majerska J., Brzezowska J., Wojdyło A., Figiel A. (2020). The Influence of Maltodextrin and Inulin on the Physico-Chemical Properties of Cranberry Juice Powders. ChemEngineering.

[19] Ruszkowska M., Kropisz P., Wiśniewska Z. (2019). Evaluation of the stability of the storage of selected fruit and vegetables freeze-dried powder based on the characteristics of the sorption properties. Sci. J. Gdyn. Marit..

[20] Butkevičiūtė A., Liaudanskas M., Ramanauskienė K., Janulis V. (2021). Biopharmaceutical Evaluation of Capsules with Lyophilized Apple Powder. Molecules.

[21] Hernández-González S.I., García-Castañeda J.I., Alba-Romero J.J., Martínez-Romero A., Chew-Madinaveitia R.G., Ortega-Sanchez J.L. (2021). Manufacture of Hard Gelatin Capsules from a Lyophilisate of the Morus Nigra Fruit. SJMH..

[22] Baeza R., Sánchez V., Salierno G., Molinari F., López P., Chirife J. (2021). Storage Stability of Anthocyanins in Freeze-Dried Elderberry Pulp Using Low Proportions of Encapsulating Agents. Food Sci. Technol. Int..

[23] Lee J. (2016). Anthocyanin Analyses of Vaccinium Fruit Dietary Supplements. Food Sci. Nutr..

[24] Du Y., Zhang M., Mujumdar A.S., Liu W., Yang C. (2022). Innovative Applications of Freeze-Drying to Produce Compound Formula Instant Foods: A Review. Dry. Technol..

[25] Grace M.H., Massey A.R., Mbeunkui F., Yousef G.G., Lila M.A. (2012). Comparison of Health-Relevant Flavonoids in Commonly Consumed Cranberry Products. J. Food Sci..

[26] Renaud V., Faucher M., Perreault V., Serre E., Dubé P., Boutin Y., Bazinet L. (2020). Evolution of Cranberry Juice Compounds during in Vitro Digestion and Identification of the Organic Acid Responsible for the Disruption of in Vitro Intestinal Cell Barrier Integrity. J. Food Sci. Technol..

[27] Fernandes I., Faria A., Calhau C., de Freitas V., Mateus N. (2014). Bioavailability of Anthocyanins and Derivatives. J. Funct. Foods.

[28] Hair R., Sakaki J.R., Chun O.K. (2021). Anthocyanins, Microbiome and Health Benefits in Aging. Molecules.

[29] Prasain J.K., Grubbs C., Barnes S. (2020). Cranberry Anti-Cancer Compounds and Their Uptake and Metabolism: An Updated Review. J. Berry Res..

[30] Jayarathne S., Stull A.J., Park O., Kim J.H., Thompson L., Moustaid-Moussa N. (2019). Protective Effects of Anthocyanins in Obesity-Associated Inflammation and Changes in Gut Microbiome. Mol. Nutr. Food Res..

[31] Shen Y., Zhang N., Tian J., Xin G., Liu L., Sun X., Li B. (2022). Advanced Approaches for Improving Bioavailability and Controlled Release of Anthocyanins. J. Control. Release.

[32] Chaudhari S.P., Patil P.S. (2012). Pharmaceutical Excipients: A Review. Int. J. Adv. Pharm. Biol. Chem..

[33] Anpilova A.Y., Mastalygina E.E., Khrameeva N.P., Popov A.A. (2020). Methods for Cellulose Modification in the Development of Polymeric Composite Materials (Review). Russ. J. Phys. Chem. B.

[34] Eloy J.O., Marchetti J.M. (2014). Solid Dispersions Containing Ursolic Acid in Poloxamer 407 and PEG 6000: A Comparative Study of Fusion and Solvent Methods. Powder Technol..

[35] Lin L., Mao X., Sun Y., Cui H. (2018). Antibacterial Mechanism of Artemisinin/Beta-Cyclodextrins against Methicillin-Resistant Staphylococcus Aureus (MRSA). Microb. Pathog..

[36] Spears J.K., Karr-Lilienthal L.K., Fahey G.C. (2005). Influence of Supplemental High Molecular Weight Pullulan or γ-Cyclodextrin on Ileal and Total Tract Nutrient Digestibility, Fecal Characteristics, and Microbial Populations in the Dog. Arch. Anim. Nutr..

[37] Illum L. (1998). Chitosan and Its Use as a Pharmaceutical Excipient. Pharm. Res..

[38] Dodane V., Vilivalam V.D. (1998). Pharmaceutical Applications of Chitosan. Fharm. Sci. Technol..

[39] Shipp J., Abdel-Aal E.-S.M. (2010). Food Applications and Physiological Effects of Anthocyanins as Functional Food Ingredients. Open Food Sci. J..

[40] Guo X., Yang B., Tan J., Jiang J., Li D. (2016). Associations of Dietary Intakes of Anthocyanins and Berry Fruits with Risk of Type 2 Diabetes Mellitus: A Systematic Review and Meta-Analysis of Prospective Cohort Studies. Eur. J. Clin. Nutr..

[41] Brown P.N., Murch S.J., Shipley P. (2012). Phytochemical Diversity of Cranberry (*Vaccinium Macrocarpon* Aiton) Cultivars by Anthocyanin Determination and Metabolomic Profiling with Chemometric Analysis. J. Agric. Food Chem..

[42] Xue H., Tan J., Li Q., Cai X., Tang J. (2021). Optimization Ultrasound-assisted Extraction of Anthocyanins from Cranberry Using Response Surface Methodology Coupled with Genetic Algorithm and Identification Anthocyanins with HPLC-MS 2. J. Food Process. Preserv..

[43] Narwojsz A., Tańska M., Mazur B., Borowska E.J. (2019). Fruit Physical Features, Phenolic Compounds Profile and Inhibition Activities of Cranberry Cultivars (*Vaccinium macrocarpon*) Compared to Wild-Grown Cranberry (*Vaccinium oxycoccus*). Plant Foods Hum. Nutr..

[44] Mallik J., Faruq A.A., Chowdhury H.B., Dinar A.M. (2013). Hard Gelatin Capsules (Two Piece)—A Unique Pharmaceutical Dosage Form—An Exhaustive Review. Asian J. Pharm. Res..

[45] Guo M., Muller F.X., Augsburger L.L. (2002). Evaluation of the Plug Formation Process of Silicified Microcrystalline Cellulose. Int. J. Pharm..

[46] Kamel S., Ali N., Jahangir K., Shah S.M., El-Gendy A.A. (2008). Pharmaceutical Significance of Cellulose: A Review. Express Polym. Lett..

[47] Mazzaracchio P., Pifferi P., Kindt M., Munyaneza A., Barbiroli G. (2004). Interactions between Anthocyanins and Organic Food Molecules in Model Systems. Int. J. Food Sci. Technol..

[48] Klavins L., Kviesis J., Klavins M. (2017). Comparison of Methods of Extraction of Phenolic Compounds from American Cranberry (*Vaccinium Macrocarpon* L.) Press Residues. Agron. Res..

[49] Herrera-Balandrano D.D., Chai Z., Beta T., Feng J., Huang W. (2021). Blueberry Anthocyanins: An Updated Review on Approaches to Enhancing Their Bioavailability. Trends Food Sci. Technol..

[50] Rahman S., Hasan S., Nitai A.S., Nam S., Karmakar A.K., Ahsan S., Shiddiky M.J.A., Ahmed M.B. (2021). Recent Developments of Carboxymethyl Cellulose. Polymers.

[51] Roy J., Ferri A., Giraud S., Jinping G., Salaün F. (2018). Chitosan–Carboxymethylcellulose-Based Polyelectrolyte Complexation and Microcapsule Shell Formulation. Int. J. Mol. Sci..

[52] Del Valle E.M.M. (2004). Cyclodextrins and Their Uses: A Review. Process Biochem..

[53] Hui B.Y., Raoov M., Zain N.N.M., Mohamad S., Osman H. (2017). Combination of Cyclodextrin and Ionic Liquid in Analytical Chemistry: Current and Future Perspectives. Crit. Rev. Anal. Chem..

[54] Fernandes A., Rocha M.A.A., Santos L.M.N.B.F., Brás J., Oliveira J., Mateus N., de Freitas V. (2018). Blackberry Anthocyanins: β-Cyclodextrin Fortification for Thermal and Gastrointestinal Stabilization. Food Chem..

[55] Ge J., Yue P., Chi J., Liang J., Gao X. (2018). Formation and Stability of Anthocyanins-Loaded Nanocomplexes Prepared with Chitosan Hydrochloride and Carboxymethyl Chitosan. Food Hydrocoll..

[56] Alfaro-Viquez E., Esquivel-Alvarado D., Madrigal-Carballo S., Krueger C.G., Reed J.D. (2018). Cranberry Proanthocyanidin-Chitosan Hybrid Nanoparticles as a Potential Inhibitor of Extra-Intestinal Pathogenic Escherichia Coli Invasion of Gut Epithelial Cells. Int. J. Biol. Macromol..

[57] Călinoiu L.-F., Ştefănescu B., Pop I., Muntean L., Vodnar D. (2019). Chitosan Coating Applications in Probiotic Microencapsulation. Coatings.

[58] da Silva Carvalho A.G., da Costa Machado M.T., de Freitas Queiroz Barros H.D., Cazarin C.B.B., Maróstica Junior M.R., Hubinger M.D. (2019). Anthocyanins from Jussara (*Euterpe Edulis* Martius) Extract Carried by Calcium Alginate Beads Pre-Prepared Using Ionic Gelation. Powder Technol..

[59] Sonia T.A., Sharma C.P., Jayakumar R.I., Prabaharan M., Muzzarelli R.A.A. (2011). Chitosan and Its Derivatives for Drug Delivery Perspective. Chitosan and Its Derivatives for Drug Delivery Perspective.

[60] He B., Ge J., Yue P., Yue X., Fu R., Liang J., Gao X. (2017). Loading of Anthocyanins on Chitosan Nanoparticles Influences Anthocyanin Degradation in Gastrointestinal Fluids and Stability in a Beverage. Food Chem..

[61] Ge J., Yue X., Wang S., Chi J., Liang J., Sun Y., Gao X., Yue P. (2019). Nanocomplexes Composed of Chitosan Derivatives and β-Lactoglobulin as a Carrier for Anthocyanins: Preparation, Stability and Bioavailability in Vitro. Food Res. Int..

[62] Wang M., Zhang Z., Sun H., He S., Liu S., Zhang T., Wang L., Ma G. (2022). Research Progress of Anthocyanin Prebiotic Activity: A Review. Phytomedicine.

[63] Liu J., Hao W., He Z., Kwek E., Zhu H., Ma N., Ma K.Y., Chen Z.-Y. (2021). Blueberry And Cranberry Anthocyanin Extracts Reduce Bodyweight and Modulate Gut Microbiota in C57BL/6 J Mice Fed with a High-Fat Diet. Eur. J. Nutr..

[64] Anhê F.F., Roy D., Pilon G., Dudonné S., Matamoros S., Varin T.V., Garofalo C., Moine Q., Desjardins Y., Levy E. (2015). A Polyphenol-Rich Cranberry Extract Protects from Diet-Induced Obesity, Insulin Resistance and Intestinal Inflammation in Association with Increased Akkermansia Spp. Population in the Gut Microbiota of Mice. Gut.

[65] Jamar G., Estadella D., Pisani L.P. (2017). Contribution of Anthocyanin-Rich Foods in Obesity Control through Gut Microbiota Interactions: Anthocyanin-Rich Foods in Obesity Control. BioFactors.

[66] (2019). Monograph: 2.2.32. Loss on Drying.

[67] Re R., Pellegrini N., Proteggente A., Pannala A., Yang M., Rice-Evans C. (1999). Antioxidant activity applying an improved ABTS radical cation decolorization assay. Free Radic. Biol. Med..

[68] Raudone L., Vilkickyte G., Pitkauskaite L., Raudonis R., Vainoriene R., Motiekaityte V. (2019). Antioxidant Activities of *Vaccinium vitis-idaea* L. Leaves within Cultivars and Their Phenolic Compounds. Molecules.

[69] Raudone L., Raudonis R., Liaudanskas M., Janulis V., Viskelis P. (2017). Phenolic antioxidant profiles in the whole fruit, flesh and peel of apple cultivars grown in Lithuania. Sci. Hortic..

[70] (2007). Monograph: 2.9.5. Uniformity of Mass of Single-Dose Preparations.

[71] (2007). Monograph: 2.9.1. Disintegration of Tablets and Capsules.

[72] AlMajed Z., Salkho N.M., Sulieman H., Husseini G.A. (2022). Modeling of the In Vitro Release Kinetics of Sonosensitive Targeted Liposomes. Biomedicines.

[73] Vilkickyte G., Motiekaityte V., Vainoriene R., Liaudanskas M., Raudone L. (2021). Development, validation, and application of UPLC-PDA method for anthocyanins profiling in *Vaccinium* L. berries. J. Berry Res..

